# First steps towards diagnosing long COVID

**DOI:** 10.1016/j.ebiom.2022.104306

**Published:** 2022-10-12

**Authors:** eBioMedicine

At this point in the COVID-19 pandemic, some might say we have all the necessary tools at hand to begin resuming a normal life. Indeed, we now have several excellent vaccines, effective treatments, antivirals, and the ability to determine at home—with a 15 min antigen test—whether we might be infectious to others. While these tools can (and should) help to reduce our collective anxiety about the pandemic, a percentage of those who were infected by the SARS-CoV-2 virus would say that life has not returned to normalcy. According to an updated pulse survey conducted between July 27 and Aug 8, 2022, by the US Census Bureau in collaboration with the National Center for Health Statistics, an estimated 17% of all previously-infected adults in the US are currently experiencing some form (one or more symptoms) of long-term sequelae more than 4 weeks (and some many months) after an infection with SARS-CoV-2. Recent analyses conducted for the WHO by the Institute for Health Metrics and Evaluation estimated that more than 17 million individuals have been affected by long COVID in the WHO European region alone. Some have reported that symptoms are severe enough that they are unable to carry out daily activities. By any estimate, many millions of people around the globe have reported long-term effects from COVID, and many remain unwell despite the tools we have available to manage the acute phase of SARS-CoV-2 infections. Unfortunately, new variants of the virus continue to emerge, and with new infections we will likely continue to see new cases of long COVID (also known as post-acute SARS-CoV-2 infection [PASC]). Yet for a condition that remains this prevalent—and debilitating for some—we are still unable to unambiguously diagnose or effectively treat PASC for many patients.

PASC can affect multiple organs and systems, can vary symptomatically between individuals, and can even manifest differently over time within the same person. Separate individuals might use synonymous terms to describe the same symptoms, further complicating efforts towards unified terminology and diagnostic guidelines. In an effort towards standardising descriptions of PASC symptoms, in a December 2021 *eBioMedicine* study, Robinson and colleagues curated electronic health data from 81 published PASC cohorts and mapped 287 unique clinical findings to standardised Human Phenotype Ontology terms. Using a common language to describe the diverse manifestations of PASC should help clinicians document and compare symptom data, and supports the development of robust algorithms able to extract and analyse PASC data from electronic health records (EHRs).

Data derived from EHRs have proven to be an important resource for PASC researchers. In a study from the July 2022 issue of *The Lancet Digital Health*, authors examined de-identified EHR data, including demographics, medical conditions, and drug orders for patients from the National COVID Cohort Collaborative (N3C) dataset, which is sponsored by the National Center for Advancing Translational Sciences and part of the larger National Institutes of Health Recover Initiative. By using the N3C data and comparison data from three separate long COVID clinics, the authors were able to train machine learning models to look for patterns that could help predict whether a person might be diagnosed with PASC. Their models achieved high accuracy, with area under the receiver operator curve values of 0·92 (for all patients), 0·90 (for hospitalised patients), and 0·85 (for non-hospitalised patients). Machine learning models such as these will be useful for clinicians for identifying patients at risk of PASC who might need specialty care, or who qualify for participation in PASC-related clinical trials.

Although machine learning tools could represent one useful option for guiding diagnoses, researchers are also working to identify PASC biomarkers and to develop improved blood tests to help support decision making in the clinic. In some instances, a patient could present with symptoms consistent with PASC, but might not know whether they were infected with SARS-CoV-2, or the patient might not have obtained a confirmatory PCR or antigen test. Although standard COVID-19 tests have the potential to be highly accurate, sensitivity is dependent in part on timing, and in most instances the tests must be done during the acute phase of SARS-CoV-2 infection. To help identify markers of previous SARS-CoV-2 infection in patients who are seronegative, authors of a study in the July 2022 issue of *eBioMedicine* examined memory T-cell responses in a cohort of patients with confirmed infection or long COVID and compared them with samples collected before the pandemic. They found that stimulating T cells with SARS-CoV-2 viral peptides resulted in the production of interleukin (IL)-2, and that their assay was able to discriminate between those with a confirmed infection (the infections in the study were acquired between April and October, 2020) and an unexposed control group. Given the wide diversity of PASC symptoms and potential overlap with other diseases and conditions, determining previous SARS-CoV-2 infection in a patient's history could represent an important step in confirming a PASC diagnosis.

In addition to diagnostics, researchers are on the hunt for blood biomarkers and immune profiles that could help to track PASC disease trajectories and help us to uncover mechanisms of disease. A study posted on the medRxiv preprint server (posted in August 2022) from the Putrino and Iwasaki laboratories reported on several immunological and hormone factors in a cross-sectional study of 215 individuals. The authors found that reduced amounts of the hormone cortisol were most strongly predictive of long COVID status. Although the study was exploratory and has not yet been peer-reviewed, authors proposed that a set of factors, including cortisol, IL-8, and galectin-1, could be worth further testing as specific biomarkers for PASC. There were several other intriguing correlations with long COVID status, including an increase in antibody responses to latent herpesviruses, such as Epstein Barr virus, which may hint that viral reactivation could potentially be linked to the disease. Although these findings need to be validated in larger cohorts, the study presents a wealth of data that could provide important clues into potential pathways and markers of PASC.

Although the mechanisms of PASC are still not clear, researchers have begun setting a strong foundation for studying PASC pathology with improved tools for diagnosing and tracking the disease. These developments are an important first step towards both identifying eligible patients and for monitoring responses in clinical trials for new therapeutic interventions. At *eBioMedicine*, we support research that helps us to better understand and treat PASC, and acknowledge the sense of urgency felt by those affected.

